# 马立巴韦相比缬更昔洛韦治疗移植后巨细胞病毒感染的疗效和安全性的Ⅲ期、多中心、随机、双盲、阳性对照研究：AURORA试验中国人群亚组分析

**DOI:** 10.3760/cma.j.cn121090-20250626-00300

**Published:** 2025-12

**Authors:** 丽 宣, 河 黄, 兰平 许, 粤文 符, 峰 陈, 荧 王, 顺清 王, 琳 董, 静怡 吴, 德沛 吴

**Affiliations:** 1 南方医科大学南方医院，广州 510515 Nanfang Hospital, Southern Medical University, Guangzhou 510515, China; 2 浙江大学医学院附属第一医院，杭州 310003 The First Affiliated Hospital, Zhejiang University School of Medicine, Hangzhou 310003, China; 3 北京大学人民医院，北京 100044 Peking University People's Hospital, Beijing 100044, China; 4 河南省肿瘤医院，郑州 450008 Henan Cancer Hospital, Zhengzhou 450008, China; 5 苏州大学附属第一医院，苏州 215006 The First Affiliated Hospital of Soochow University, Suzhou 215006, China; 6 广州市第一人民医院，广州 510180 Guangzhou First People's Hospital, Guangzhou 510180, China; 7 武田亚太生物医药研发有限公司，上海 200126 Takeda Asia-Pacific Biopharmaceutical R&D Co., Ltd., Shanghai 200126, China; 8 武田（中国）国际贸易有限公司，上海 200126 Takeda（China）International Trading Co., Ltd., Shanghai 200126, China

**Keywords:** 巨细胞病毒, 造血干细胞移植, 马立巴韦, 缬更昔洛韦, Cytomegalovirus, Hematopoietic stem cell transplantation, Maribavir, Valganciclovir

## Abstract

**目的:**

比较马立巴韦与缬更昔洛韦在造血干细胞移植（HSCT）受者中治疗无症状巨细胞病毒（CMV）感染的疗效和安全性。

**方法:**

这项Ⅲ期、多中心、随机、双盲、阳性对照研究将HSCT后首次发生的无症状CMV感染的参与者按1∶1的比例随机接受马立巴韦（400 mg，每日2次）或缬更昔洛韦（450 mg，每日1次至900 mg每日2次，剂量基于肌酐清除率调整）治疗8周，随访时间为研究第8～20周。主要疗效终点是在研究第8周结束时确认的CMV病毒血症清除。安全性评价包括治疗期间出现的不良事件（TEAE）和严重不良事件（SAE）。

**结果:**

共随机入组18例来自中国亚组人群的参与者，马立巴韦组和缬更昔洛韦组各9例参与者。在第8周时，马立巴韦组和缬更昔洛韦组均有77.8％的参与者达到了确认的CMV病毒血症清除，未校正差异（马立巴韦对缬更昔洛韦）为0.0％（95％ *CI*: −38.4％, 38.4％）。马立巴韦组和缬更昔洛韦组在第8周达到CMV血症清除并维持至第16周的应答者比例分别为44.4％和55.6％，未校正差异为−11.1％（95％ *CI*: −57.0％, 34.8％）。马立巴韦组和缬更昔洛韦组治疗观察期间至少发生1起TEAE的参与者比例均为100％，因TEAE导致停用研究药物的参与者比例分别为11.1％和33.3％，治疗相关SAE发生率分别为0和22.2％。马立巴韦组在治疗观察期间中性粒细胞减少症发生率在数值上低于缬更昔洛韦组（22.2％对55.6％）。

**结论:**

在中国人群中，马立巴韦治疗无症状CMV感染的HSCT受者中清除CMV病毒血症方面与缬更昔洛韦相当，且具有可接受的安全性和良好的耐受性，特别是中性粒细胞减少症的发生率低于缬更昔洛韦组。

## Abstract (en)

**Objective:**

This study aimed to compare the efficacy and safety of maribavir and valganciclovir in the treatment of asymptomatic cytomegalovirus（CMV）infection in hematopoietic stem cell transplant（HSCT）recipients.

**Methods:**

In this phase 3, multicenter, randomized, double-blind, positive-controlled study, subjects with first episode of asymptomatic CMV infection post-HSCT were randomly assigned in a 1∶1 ratio to receive maribavir（400 mg, twice daily）or valganciclovir (dose-adjusted for renal clearance）for 8 weeks with a follow-up period spanned from 8 to week 20. The primary efficacy endpoint includes confirmed CMV viremia clearance at 8 weeks. Safety assessment included treatment-emergent adverse events（TEAEs）and serious adverse events（SAEs）.

**Results:**

The Chinese population subgroup included 18 subjects, comprising 9 subjects each in the maribavir and valganciclovir arms. At 8 weeks, 77.8％ of the subjects in both arms achieved confirmed CMV viremia clearance, with an unadjusted difference（maribavir *vs* valganciclovir）of 0.0％（95％ *CI*: −38.4％ to 38.4％）. The proportions of responders who achieved CMV viremia clearance at 8 weeks and maintained it until 16 weeks were 44.4％ and 55.6％ in the maribavir and valganciclovir arms, respectively, with an unadjusted difference of −11.1％（95％ *CI*: −57.0％ to 34.8％）. During the treatment observation period, 100％ of subjects in both the maribavir and valganciclovir arms experienced at least one TEAE, causing 11.1％ and 33.3％ of subjects, respectively, to discontinue the study drug. The incidence of treatment-related SAEs was 0 and 22.2％ in the maribavir and valganciclovir arms, respectively. Further, the incidence of neutropenia during the treatment observation period was lower in the maribavir arm than in the valganciclovir arm（22.2％ *vs* 55.6％）.

**Conclusion:**

In the Chinese population, maribavir may be comparable to valganciclovir in clearing CMV viremia to treat HSCT recipients with asymptomatic CMV infection, with acceptable safety and good tolerability, especially being numerically lower than valganciclovir in terms of neutropenia.

巨细胞病毒（CMV）感染是接受造血干细胞移植（HSCT）的患者常见的并发症，中国人群异基因HSCT（allo-HSCT）后CMV感染发生率达50％[1]–[3]，全因死亡率接近30％[3]。发生CMV感染的患者移植植入失败的风险增加2倍，死亡风险增加3倍[4]–[9]。现有的抗CMV治疗药物包括更昔洛韦、缬更昔洛韦、膦甲酸钠和西多福韦[10]。这些药物用于HSCT患者CMV感染抢先治疗的有效率在62.0％～89.5％[11]–[14]，但受限于其治疗相关毒性[15]–[17]，通常需要在治疗过程中调整剂量或中断治疗，进而导致CMV感染复发并增加患者的全因死亡率[18]–[19]。此外，抗CMV药物治疗相关毒性的应对和管理措施对患者和医疗保健系统都造成了沉重的负担[18],[20]。因此，需要一种疗效相当且耐受性更好的新药物来控制HSCT受者中发生的CMV感染。

马立巴韦是一种口服的苯并咪唑核苷类抗病毒药物，通过抑制CMV特异性UL97蛋白激酶来抑制CMV DNA复制、衣壳化和病毒衣壳的核逃逸，从而具有多模态抗CMV活性[21]。马立巴韦已被证实对CMV具有体外抗病毒活性，其中包括对更昔洛韦、膦甲酸钠或西多福韦有耐药性的病毒株[22]。在一项针对移植后难治性CMV感染的关键Ⅲ期临床试验中，接受马立巴韦（400 mg，每日2次）持续8周治疗的患者，其第8周的CMV病毒血症清除率优于研究者指定的常规抗CMV治疗，且这一临床获益持续维持至第16周。安全性方面，马立巴韦组因治疗期间出现的不良事件（TEAE）中断治疗的患者人数少于常规治疗组[15]。在类似患者群体中进行的Ⅱ期研究表明，长达24周的马立巴韦治疗方案具有抗CMV的疗效并且安全性和耐受性符合预期[23]。在传统抗CMV药物常见治疗相关毒性方面，马立巴韦在多项临床试验中显示出更好的耐受性[15],[23]–[27]。基于以上研究结果，马立巴韦于2023年12月19日在我国获得批准用于治疗HSCT或实体器官移植后CMV感染和（或）疾病，且对一种或多种既往治疗（更昔洛韦、缬更昔洛韦、西多福韦或膦甲酸钠）难治（伴或不伴基因型耐药）的成人患者[28]。马立巴韦在移植后首次发生CMV感染中的疗效也已在临床试验中得到探索。一项针对HSCT和实体器官移植后首次发生CMV感染的患者的Ⅱ期研究表明剂量为400 mg、800 mg和1 200 mg每日2次的马立巴韦长达12周的治疗方案在CMV血症清除方面的效果与缬更昔洛韦相似，且具有更低的中性粒细胞减少症发生率[24]。

AURORA研究（NCT02927067）是一项比较马立巴韦与缬更昔洛韦对HSCT后首次发生的无症状CMV感染的疗效和安全性的Ⅲ期、多中心、随机、双盲、双模拟、阳性药物对照研究。该研究对全球97家中心547例患者的总体分析结果显示，马立巴韦在主要终点方面未达到预设的非劣效性界值（7.0％）要求，未能证明其非劣效于缬更昔洛韦。但马立巴韦在维持CMV血症清除率方面表现出与缬更昔洛韦相当的疗效。此外，马立巴韦组的TEAE发生率较低，特别是因中性粒细胞减少而导致治疗中断的病例数更少[25]。

本研究是AURORA研究在中国人群中的亚组分析，在中国人群HSCT受者首次发生无症状CMV感染时，比较马立巴韦相对于缬更昔洛韦在CMV血症清除方面的疗效和安全性。

## 资料与方法

1. 研究设计与患者选择：AURORA试验的研究方法已发表[25]。入选标准包括年龄≥16岁、预期寿命≥8周、在HSCT后首次记录的无症状CMV血症（原发性或再激活）。病毒血症的确认需满足以下实验室标准：由当地或中心实验室通过间隔至少1 d的连续2次检测证实全血CMV DNA载量在1 365～273 000 IU/ml，或血浆CMV DNA载量在455～91 000 IU/ml。根据研究者的评估，无症状CMV感染被定义为未出现组织侵袭性CMV疾病的感染[29]。患者入组时必须符合ANC≥ 1 000/mm^3^、HGB≥80 g/L和PLT≥25 000/mm^3^。

排除标准包括研究者评估的组织侵袭性疾病，已知对更昔洛韦、缬更昔洛韦、膦甲酸钠或西多福韦具有基因型耐药的CMV感染或复发性CMV血症（在移植后至少发生1次先前记录的CMV感染以及在2次感染之间至少2周检测不出CMV DNA）。此外，以下患者也不符合入选条件：①已接受更昔洛韦、缬更昔洛韦或膦甲酸钠治疗当前CMV感染超过72 h者；②研究治疗开始前30 d内使用过来特莫韦或其他试验性抗CMV药物者；③既往接种过CMV疫苗者；④当前正在接受来氟米特或青蒿琥酯治疗者。

入选的患者以1∶1的比例随机接受400 mg每日2次剂量的马立巴韦或450 mg每日1次至900 mg每日2次剂量的缬更昔洛韦（根据肾功能调整）口服给药治疗，治疗8周后再进行12周随访。评估参与者治疗过程中的中性粒细胞减少情况，并可根据ANC水平调整缬更昔洛韦的剂量。根据基线前最后一次测得的全血或血浆CMV DNA浓度以及是否存在急性移植物抗宿主病（aGVHD）对参与者进行分层。盲法一直维持到最后一例患者末次访视后数据库锁定为止。

本研究根据国际协调会议（ICH）制定的《药物临床试验质量管理规范（GCP）》和《赫尔辛基宣言》的原则进行。各研究中心的机构审查委员会/独立伦理委员会批准了本研究。独立数据监测委员会定期审查所有研究数据。所有患者/法定监护人均签署了书面知情同意书。

2. 疗效与安全性评价标准：于研究第8周时评估CMV血症清除，在中心实验室使用COBAS® AmpliPrep/COBAS® TaqMan® CMV Test进行检测，以控制不同实验室检测方法差异导致的变异性。主要疗效终点是在研究第8周结束时确认的CMV血症清除。无论病毒载量如何，在研究第8周前接受替代、非研究抗CMV治疗的参与者均被视为无应答者。关键次要终点是在研究第8周结束时达到病毒血症清除且无组织侵袭性CMV病，并且在停止治疗后该疗效维持至研究第16周。安全性评价包括TEAE和治疗期间出现的严重不良事件（SAE）。

3. 统计学处理：主要疗效分析基于调整的随机化集，支持性分析基于符合方案（Per-protocol, PP）集。对于二元终点（应答者或非应答者），使用Cochran-Mantel-Haenszel（CMH）加权平均值获得各分层治疗组间应答者比例的差异，并使用CMH方法进行检验，以基线血浆中的CMV DNA浓度水平和是否存在aGVHD作为分层因素。由于中国亚组人群中入组的参与者例数非常有限（马立巴韦组9例参与者和缬更昔洛韦组9例参与者），本文仅列出每个疗效终点的描述性总结，以及未校正差异（95％ *CI*）。

安全性分析采用安全集作为分析人群，该人群包括所有接受至少1剂研究药物治疗的参与者。除非特别说明，所有安全性评估均基于治疗期间及整个研究期间收集的数据。分析方法采用描述性统计，按治疗组别汇总报告各项安全性终点指标。需要特别说明的是，本研究未对各治疗组间的安全性终点进行假设检验。

## 结果

1. 参与者基本信息：在AURORA研究的中国亚组分析中，随机化集包括2021年5月至2022年6月来自中国6家研究中心的18例参与者，其中有13例参与者［马立巴韦组77.8％（7/9）参与者，缬更昔洛韦组66.7％（6/9）参与者］完成了8周的研究指定治疗。马立巴韦组和缬更昔洛韦组中治疗终止的原因包括：不良事件（分别为0例和3例）、缺乏疗效（分别为1例和0例）和参与者状况不佳（分别为1例和0例）。两个治疗组均未报告因死亡导致的治疗终止（图1）。两组参与者的人口学和基线特征相当（表1）。基线定义为研究治疗首次给药日期或未接受研究治疗的参与者的随机化日期当天或之前的末次值。

**图1:**
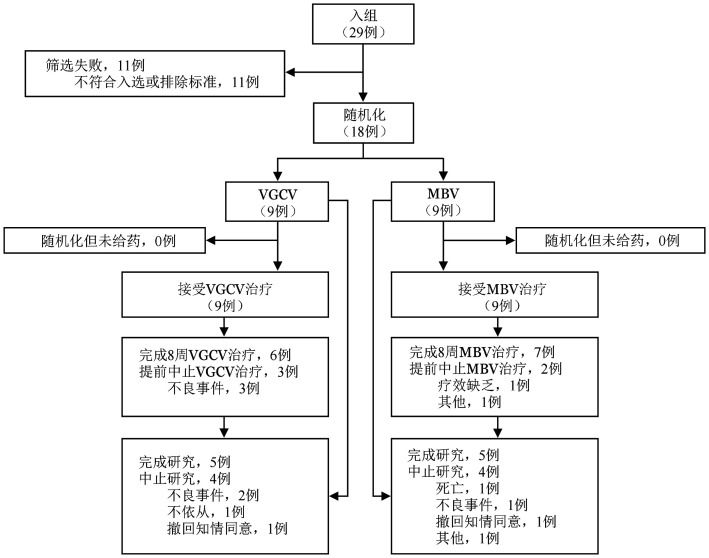
AURORA研究的中国亚组分析参与者的入组、随机化和随访情况流程图 **注** MBV：马立巴韦；VGCV：缬更昔洛韦；百分比基于随机化参与者的数量

**表1:** AURORA研究的中国亚组分析参与者人口学和基线特征

特征	缬更昔洛韦（9例）	马立巴韦（9例）
年龄（岁，*x*±*s*）	32.4±12.4	30.8±10.6
年龄分类［例（％）］		
>><18岁	1（11.1）	0（0）
18～44岁	5（55.6）	8（88.9）
45～64岁	3（33.3）	1（11.1）
性别［例（％）］		
男性	6（66.7）	5（55.6）
女性	3（33.3）	4（44.4）
CMV DNA水平［IU/ml，*M*（范围）］^a^	3 870（69～23 200）	20 700（1 960～767 000）
CMV DNA水平分类［例（％）］^a^		
极低（455 ～ <910 IU/ml）	1（11.1）	0（0）
偏低（910 ～ <9 100 IU/ml）	5（55.6）	3（33.3）
高（≥9 100 IU/ml）	3（33.3）	6（66.7）
急性GVHD［例（％）］		
无	8（88.9）	8（88.9）
有	1（11.1）	1（11.1）
慢性GVHD［例（％）］		
无	9（100）	9（100）
有	0（0）	0（0）
预处理方案［例（％）］		
清髓性	7（77.8）	7（77.8）
非清髓性	1（11.1）	0（0）
减低强度预处理方案	1（11.1）	2（22.2）
CMV血清状态［例（％）］		
供者阳性/受者阳性	6（66.7）	0（0）
供者阴性/受者阴性	3（33.3）	7（77.8）
缺失	0（0）	2（22.2）
使用抗胸腺细胞球蛋白［例（％）］		
是	9（100）	9（100）
否	0（0）	0（0）
既往HSCT次数［例（％）］		
0	7（77.8）	9（100）
1	0（0）	0（0）
2	2（22.2）	0（0）
当前移植的原因［例（％）］		
急性髓系白血病	7（77.8）	5（55.6）
慢性髓细胞性白血病	1（11.1）	0（0）
急性淋巴细胞白血病	0（0）	2（22.2）
骨髓异常增生综合征	0（0）	1（11.1）
其他	1（11.1）	1（11.1）
异基因HSCT［例（％）］	9（100）	9（100.0）
供者类型［例（％）］		
同胞相合供者	1（11.1）	0（0）
HLA匹配的其他亲属	6（66.7）	4（44.4）
HLA不匹配亲属	2（22.2）	4（44.4）
无关供者	0（0）	1（11.1）
CMV预防治疗［例（％）］		
否	9（100）	7（77.8）
是	0（0）	2（22.2）
当前移植至研究首次给药时间［d，*M*（范围）］	40（33～79）	56（31～76）

**注** CMV：巨细胞病毒；GVHD：移植物抗宿主病；HSCT：造血干细胞移植；HLA：人白细胞抗原。^a^：为基线时中心实验室的血浆CMV DNA水平

2. 主要疗效终点：第8周时，马立巴韦组和缬更昔洛韦组各有7例（77.8％）参与者达到了确认的CMV血症清除，应答者比例的未校正差异（马立巴韦对缬更昔洛韦）为0.0％（95％ *CI*：−38.4％～38.4％）（表2）。

**表2:** 按治疗组列出的研究第8周时确认的病毒血症清除应答（调整的随机化集）

确认的病毒血症清除应答	缬更昔洛韦（9例）	马立巴韦（9例）
应答者	7（77.8）	7（77.8）
无应答者	2（22.2）	2（22.2）
应答者比例的未校正差异^a^	0.0％（95％*CI*：−38.4％～38.4％）	
应答者比例的校正后差异^b^	13.0％（95％*CI*：−28.1％～54.2％）	

**注** 研究第8周结束时确认病毒血症清除的参与者为应答者（即使药物治疗时间不足8周），开始禁用抗巨细胞病毒（CMV）药物后>1 d采集的血浆CMV DNA结果视为缺失；^a^：通过正态近似法计算未校正比例差异（马立巴韦-缬更昔洛韦）；^b^：校正急性移植物抗宿主病（GVHD）和基线血浆CMV DNA浓度后，使用CMH加权平均值法计算比例的校正后差异（马立巴韦-缬更昔洛韦）

3. 关键次要疗效终点：分析第8周时达到CMV血症清除和CMV感染症状控制并维持疗效至第16周的参与者，马立巴韦组为4例（44.4％），缬更昔洛韦组为5例（55.6％）。治疗组之间应答者比例的未校正差异为−11.1％（95％ *CI*：−57.0％～34.8％）（表3）。

**表3:** 按治疗组列出的在研究第8周达到并维持至研究第16周的确认的病毒血症清除和CMV感染症状控制应答（调整的随机化集）

确认的病毒血症清除和CMV感染症状控制应答	缬更昔洛韦（9例）	马立巴韦（9例）
应答者	5（55.6）	4（44.4）
无应答者	4（44.4）	5（55.6）
应答者比例的未校正差异（95％ *CI*）^a^	−11.1％（−57.0％, 34.8％）	
应答者比例的校正后差异（95％ *CI*）^b^	−4.3％（−58.8％, 50.1％）	

**注** ^a^：通过正态近似法计算未校正的比例差异（马立巴韦-缬更昔洛韦）；^b^：采用CMH加权平均值方法校正比例差异（马立巴韦-缬更昔洛韦）

4. 其他次要疗效终点：不论参与者是否完成8周治疗，马立巴韦组和缬更昔洛韦组第12周CMV血症清除和CMV感染症状控制的参与者数分别为5例（55.6％）和5例（55.6％），未校正差异为0.0％（95％ *CI*：−45.9％～45.9％），第20周分别为4例（44.4％）和5例（55.6％），未校正差异为−11.1％（95％ *CI*：−57.0％～34.8％）。

在完成8周治疗的参与者中，马立巴韦组有5例（55.6％）在第8周实现CMV血症清除并维持至第12周，缬更昔洛韦组为4例（44.4％），未校正差异为11.1％（95％ *CI*：−34.8％～57.0％）。在第16周和第20周，两个治疗组仍能维持效应的参与者均为4例（44.4％），未校正差异为0.0％（95％ *CI*：−45.9％～45.9％）。

在完成8周治疗并达到病毒血症清除的参与者中，马立巴韦组或缬更昔洛韦组无参与者在研究期间的任何时间出现CMV血症复发。

5. 探索性疗效终点：在接受研究方案规定的治疗后至第8周内的任意研究时间点，马立巴韦组与缬更昔洛韦组各有7例（77.8％）参与者达到确认的CMV血症清除标准。基于Kaplan-Meier估计，马立巴韦组至首次确证CMV病毒血症清除的中位时间为18.5（95％ *CI*：8.0～无法估计）d，缬更昔洛韦组中位时间为16.0（95％ *CI*：8.0～22.0）d。研究期间任意时间点首次CMV血症清除的累积概率见图2。

**图2:**
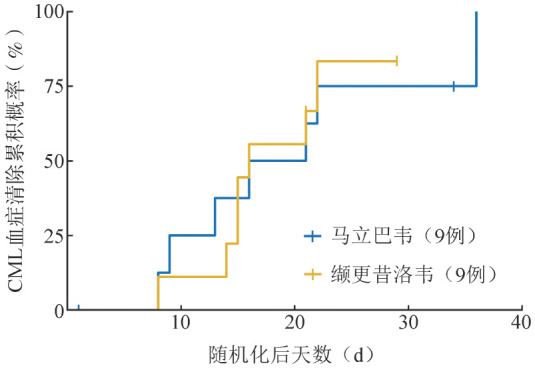
按治疗组列出的研究期间任何时间首次CMV血症清除的累积概率 **注** CMV：巨细胞病毒

6. 安全性评价：总体而言，两组均有100％的参与者在8周治疗期间出现TEAE，其中中性粒细胞减少是最常见的TEAE。在治疗观察期间，中性粒细胞减少在马立巴韦组中的发生率低于缬更昔洛韦组（22.2％对55.6％）（表4）。马立巴韦组在研究治疗期间从较低级别变为3级或4级中性粒细胞减少的比例低于缬更昔洛韦组（11.1％对44.4％）。两组在研究治疗期间各有1例（11.1％）参与者变为4级中性粒细胞减少。治疗期间，马立巴韦组和缬更昔洛韦组各有3例（33.3％）参与者需要G-CSF治疗。马立巴韦组与缬更昔洛韦组各有2例（22.2％）参与者至少接受过1种血液制品输注。其中，两组各有1例（11.1％）参与者接受了血小板输注；马立巴韦组有2例（22.2％）参与者接受了红细胞输注。

**表4:** 按系统器官分类、首选术语和治疗组列出治疗期间≥2例参与者治疗中出现的不良事件［例（％）］

系统器官分类首选术语	缬更昔洛韦（9例）	马立巴韦（9例）
任何TEAE	9（100）	9（100）
血液学不良反应	6（66.7）	4（44.4）
贫血	2（22.2）	2（22.2）
白细胞减少	4（44.4）	3（33.3）
中性粒细胞减少	5（55.6）	2（22.2）
血小板减少	2（22.2）	1（11.1）
心脏疾病	0（0）	2（22.2）
室上性心动过速	0（0）	2（22.2）
胃肠系统症状	5（55.6）	4（44.4）
齿龈出血	2（22.2）	0（0）
恶心	0（0）	3（33.3）
呕吐	1（11.1）	3（33.3）
全身性症状及给药部位各种反应	4（44.4）	2（22.2）
发热	3（33.3）	2（22.2）
免疫系统症状	4（44.4）	2（22.2）
皮肤急性移植物抗宿主病	3（33.3）	0（0）
感染及侵染类疾病	1（11.1）	7（77.8）
结膜炎	0（0）	2（22.2）
EB病毒感染	1（11.1）	2（22.2）
感染性肺炎	0（0）	2（22.2）
上呼吸道感染	1（11.1）	2（22.2）
各类检查	6（66.7）	7（77.8）
丙氨酸转氨酶升高	1（11.1）	1（11.1）
天门冬氨酸转氨酶升高	1（11.1）	1（11.1）
免疫抑制剂药物水平升高	1（11.1）	1（11.1）
中性粒细胞计数降低	4（44.4）	2（22.2）
血小板计数降低	2（22.2）	1（11.1）
尿蛋白检出	0（0）	2（22.2）
白细胞计数降低	1（11.1）	2（22.2）
代谢及营养类疾病	4（44.4）	8（88.9）
高甘油三酯血症	2（22.2）	1（11.1）
高尿酸血症	2（22.2）	0（0）
低钾血症	0（0）	4（44.4）
低蛋白血症	1（11.1）	2（22.2）

**注** TEAE：治疗期间出现的不良事件

恶心和呕吐也是常见的TEAE。马立巴韦组中，恶心（3例对0例）和呕吐（3例对1例）的发生率高于缬更昔洛韦组。

马立巴韦组中1例（11.1％）和缬更昔洛韦组中3例（33.3％）因TEAE中止治疗。两组在TEAE及导致停药的TEAE的差异主要源于中性粒细胞减少症的发生率差异，该不良事件在缬更昔洛韦组中报告频率更高。

马立巴韦治疗期间出现SAE的参与者分别为6例（66.7％）和3例（33.3％）。马立巴韦组2例（22.2％）参与者报告死亡，均被认为与研究治疗无关，而缬更昔洛韦组无参与者报告死亡。认为与研究分配的治疗相关的治疗中出现的SAE仅发生在缬更昔洛韦组（2例），分别为骨髓功能衰竭1例（11.1％）和微生态失调1例（11.1％）。

## 讨论

与AURORA全球人群基线特征相比，中国人群亚组的平均年龄较小、移植预处理方案更多采用清髓性方案、单倍体供者的比例较高，这些差异可能与中国较多采用单倍体移植相关[30]。尽管单倍体移植与较高的CMV再激活率相关，但尚无确凿证据表明供者类型对CMV抗病毒治疗的疗效存在显著影响[31]。此外，中国人群亚组中马立巴韦组的基线血浆CMV DNA中位数明显高于AURORA全球人群。这一差异可能与中国亚组样本量较小有关。真实世界研究显示较高的基线CMV DNA水平可能与马立巴韦治疗耐药以及早期病毒血症复发相关[32]，但在中国亚组中未观察到CMV DNA水平对马立巴韦疗效的影响。在本研究中，中国人群亚组中马立巴韦组的CMV血清学阴性（供者/受者均为阴性）比例达到了77.8％，而中国的实际临床实践中，CMV血清学阳性率超过90％[33]。一项对HSCT后抗CMV药物治疗临床试验的系统综述发现，移植前CMV血清学供者/受者双阳性（D+/R+）和供者阴性/受者阳性（D−/R+）患者的CMV病毒血症复发率分别为32.1％和35.8％，远高于供者/受者双阴性（D−/R−）患者的3.1％[34]。

AURORA研究因马立巴韦组中有更多的患者在8周治疗期间发生耐药以及较保守的非劣效性界值（7.0％）而未能达到其设定的非劣效主要终点，但仍有近70％接受马立巴韦治疗的移植后首次记录的无症状CMV感染患者实现了CMV血症清除[25]，其较高的病毒血症清除率与中国人群亚组的结果一致。中国人群亚组主要疗效终点分析结果显示马立巴韦组参与者在研究第8周时的CMV血症清除率与缬更昔洛韦组相当（77.8％对77.8％），而全球的总体结果显示马立巴韦组与缬更昔洛韦组在研究第8周时病毒血症清除率分别为69.6％和77.4％，校正后差异为−7.7％（95％ *CI*：−14.98％～−0.36％）[25]。中国人群亚组与全球整体结果之间在主要疗效终点上的差异可能是由于中国人群亚组较小的样本量所致。

在疗效维持方面，中国亚组的研究结果与AURORA研究的全球结果保持一致。该研究结果显示马立巴韦在12周（59.3％对57.3％，*P*＝0.606）、16周（52.7％对48.5％，*P*＝0.298）和20周（43.2％对42.3％，*P*＝0.809）时维持CMV血症清除方面与缬更昔洛韦相当[25]。在复发率方面，中国人群亚组结果显示无论马立巴韦组或缬更昔洛韦组，实现CMV血症清除的患者中均未发现研究期间CMV血症复发，而AURORA研究的全球结果显示在整个研究期间，马立巴韦组和缬更昔洛韦组CMV血症复发率分别为19.0％和22.5％[25]。与主要疗效终点结果差异类似，复发率存在差异的原因也可能是中国人群亚组样本量较小。

安全性方面，中性粒细胞减少症是HSCT后CMV感染早期患者管理的主要挑战，在亚洲HSCT受者中传统抗CMV治疗药物导致中性粒细胞减少的发生率高达30％～40％[35]。严重的中性粒细胞减少会导致被迫中断治疗、感染，甚至死亡。据统计13.6％患者因抗CMV治疗导致的严重中性粒细胞减少而中断治疗[35]–[36]。此外，中性粒细胞减少还会增加G-CSF的使用，而较长时间迁延不愈的中性粒细胞减少症会增加侵袭性真菌感染的风险，造成医疗系统的沉重负担[36]。AURORA研究全球结果显示马立巴韦组较缬更昔洛韦组中性粒细胞减少发生率更低（16.1％对52.9％），且更少的患者因中性粒细胞减少而中断治疗（4.0％对17.5％）或需要G-CSF治疗（6.2％对15.3％）[25]。在这方面，本研究中国亚组结果与AURORA研究全球结果一致。

AURORA研究的方法学具有稳定性，但研究也存在一定的局限性，例如研究方案未纳入GVHD患者的免疫抑制治疗相关数据，这与真实世界临床实践存在一定差异[25]。中国人群亚组分析仅包含18例患者样本，应谨慎解读分析结果。

总而言之，马立巴韦在维持与标准治疗疗效相当的基础上，为HSCT后首次发生CMV感染的中国患者提供了一种较为安全的新型治疗选择。在中国人群中，马立巴韦在治疗无症状CMV感染的HSCT受者中，清除CMV血症的效果与缬更昔洛韦相当，并且具有可接受的安全性和良好的耐受性，特别是中性粒细胞减少症发生率低于缬更昔洛韦组。未来还需要进一步大样本的随机对照研究或真实世界数据以验证马立巴韦治疗HSCT后无症状CMV感染在中国人群中的疗效和安全性。

## References

[1] Jakharia N, Howard D, Riedel DJ (2021). CMV Infection in Hematopoietic Stem Cell Transplantation: Prevention and Treatment Strategies[J]. Curr Treat Options Infect Dis.

[2] Ljungman P, de la Camara R, Robin C (2019). Guidelines for the management of cytomegalovirus infection in patients with haematological malignancies and after stem cell transplantation from the 2017 European Conference on Infections in Leukaemia (ECIL 7)[J]. Lancet Infect Dis.

[3] Lin R, Wu J, Liu Q (2025). Epidemiology, clinical outcomes, and treatment patterns of cytomegalovirus infection after allogeneic hematopoietic stem cell transplantation in China: a scoping review and meta-analysis[J]. Front Microbiol.

[4] Camargo JF, Kimble E, Rosa R (2018). Impact of Cytomegalovirus Viral Load on Probability of Spontaneous Clearance and Response to Preemptive Therapy in Allogeneic Stem Cell Transplantation Recipients[J]. Biol Blood Marrow Transplant.

[5] Cho S, Lee D, Kim H (2019). Cytomegalovirus Infections after Hematopoietic Stem Cell Transplantation: Current Status and Future Immunotherapy[J]. Int J Mol Sci.

[6] Robin C, Hémery F, Dindorf C (2017). Economic burden of preemptive treatment of CMV infection after allogeneic stem cell transplantation: a retrospective study of 208 consecutive patients[J]. BMC Infect Dis.

[7] Selvey LA, Lim WH, Boan P (2017). Cytomegalovirus viraemia and mortality in renal transplant recipients in the era of antiviral prophylaxis. Lessons from the western Australian experience[J]. BMC Infect Dis.

[8] Stern M, Hirsch H, Cusini A (2014). Cytomegalovirus serology and replication remain associated with solid organ graft rejection and graft loss in the era of prophylactic treatment[J]. Transplantation.

[9] Teira P, Battiwalla M, Ramanathan M (2016). Early cytomegalovirus reactivation remains associated with increased transplant-related mortality in the current era: a CIBMTR analysis[J]. Blood.

[10] Allaw F, Haddad SF, Zakhour J (2023). Management of cytomegalovirus infection in allogeneic hematopoietic stem cell transplants[J]. Int J Antimicrob Agents.

[11] Chawla JS, Ghobadi A, Mosley J (2012). Oral valganciclovir versus ganciclovir as delayed pre-emptive therapy for patients after allogeneic hematopoietic stem cell transplant: a pilot trial (04-0274) and review of the literature[J]. Transpl Infect Dis.

[12] Ljungman P, Deliliers GL, Platzbecker U (2001). Cidofovir for cytomegalovirus infection and disease in allogeneic stem cell transplant recipients. The Infectious Diseases Working Party of the European Group for Blood and Marrow Transplantation[J]. Blood.

[13] Reusser P, Einsele H, Lee J (2002). Randomized multicenter trial of foscarnet versus ganciclovir for preemptive therapy of cytomegalovirus infection after allogeneic stem cell transplantation[J]. Blood.

[14] van der Heiden PLJ, Kalpoe JS, Barge RM (2006). Oral valganciclovir as pre-emptive therapy has similar efficacy on cytomegalovirus DNA load reduction as intravenous ganciclovir in allogeneic stem cell transplantation recipients[J]. Bone Marrow Transplant.

[15] Avery RK, Alain S, Alexander BD (2022). Maribavir for Refractory Cytomegalovirus Infections With or Without Resistance Post-Transplant: Results From a Phase 3 Randomized Clinical Trial[J]. Clin Infect Dis.

[16] Ota R, Hirata A (2021). Relationship between renal dysfunction and electrolyte abnormalities in hematopoietic stem cell transplant patients treated with foscarnet[J]. J Chemother.

[17] Zavras P, Su Y, Fang J (2020). Impact of Preemptive Therapy for Cytomegalovirus on Toxicities after Allogeneic Hematopoietic Cell Transplantation in Clinical Practice: A Retrospective Single-Center Cohort Study[J]. Biol Blood Marrow Transplant.

[18] Cheng WY, Avery RK, Thompson-Leduc P (2022). Evaluation of treatment patterns, healthcare resource utilization, and costs among patients receiving treatment for cytomegalovirus following allogeneic hematopoietic cell or solid organ transplantation[J]. J Med Econ.

[19] Einsele H, Ljungman P, Boeckh M (2020). How I treat CMV reactivation after allogeneic hematopoietic stem cell transplantation[J]. Blood.

[20] De Latour RP, Chevallier P, Blaise D (2020). Clinical and economic impact of treated CMV infection in adult CMV-seropositive patients after allogeneic hematopoietic cell transplantation[J]. J Med Virol.

[21] Sun K, Fournier M, Sundberg AK (2024). Maribavir: Mechanism of action, clinical, and translational science[J]. Clin Transl Sci.

[22] Williams SL, Hartline CB, Kushner NL (2003). In vitro activities of benzimidazole D- and L-ribonucleosides against herpesviruses[J]. Antimicrob Agents Chemother.

[23] Papanicolaou GA, Silveira FP, Langston AA (2019). Maribavir for Refractory or Resistant Cytomegalovirus Infections in Hematopoietic-cell or Solid-organ Transplant Recipients: A Randomized, Dose-ranging, Double-blind, Phase 2 Study[J]. Clin Infect Dis.

[24] Maertens J, Cordonnier C, Jaksch P (2019). Maribavir for Preemptive Treatment of Cytomegalovirus Reactivation[J]. N Engl J Med.

[25] Papanicolaou GA, Avery RK, Cordonnier C (2024). Treatment for First Cytomegalovirus Infection Post-Hematopoietic Cell Transplant in the AURORA Trial: A Multicenter, Double-Blind, Randomized, Phase 3 Trial Comparing Maribavir With Valganciclovir[J]. Clin Infect Dis.

[26] Winston DJ, Saliba F, Blumberg E (2012). Efficacy and safety of maribavir dosed at 100 mg orally twice daily for the prevention of cytomegalovirus disease in liver transplant recipients: a randomized, double-blind, multicenter controlled trial[J]. Am J Transplant.

[27] Winston DJ, Young JH, Pullarkat V (2008). Maribavir prophylaxis for prevention of cytomegalovirus infection in allogeneic stem cell transplant recipients: a multicenter, randomized, double-blind, placebo-controlled, dose-ranging study[J]. Blood.

[28] 武田(中国)国际贸易有限公司 马立巴韦片说明书[Z].

[29] Ljungman P, Boeckh M, Hirsch HH (2017). Definitions of Cytomegalovirus Infection and Disease in Transplant Patients for Use in Clinical Trials[J]. Clin Infect Dis.

[30] Wang X, Huang R, Zhang X (2022). Current status and prospects of hematopoietic stem cell transplantation in China[J]. Chin Med J (Engl).

[31] Luo X, Zhu Y, Chen Y (2021). CMV Infection and CMV-Specific Immune Reconstitution Following Haploidentical Stem Cell Transplantation: An Update[J]. Front Immunol.

[32] Ni B, Wolfe CR, Arif S (2024). Real-World Experience With Maribavir for Treatment of Cytomegalovirus Infection in High-Risk Solid Organ Transplant Recipients[J]. Open Forum Infect Dis.

[33] Zuhair M, Smit GSA, Wallis G (2019). Estimation of the worldwide seroprevalence of cytomegalovirus: A systematic review and meta-analysis[J]. Rev Med Virol.

[34] Styczynski J (2018). Who Is the Patient at Risk of CMV Recurrence: A Review of the Current Scientific Evidence with a Focus on Hematopoietic Cell Transplantation[J]. Infect Dis Ther.

[35] Cho S, Ar MC, Machado CM (2023). Epidemiology, treatment patterns, and disease burden of cytomegalovirus in hematopoietic cell transplant recipients in selected countries outside of Europe and North America: A systematic review[J]. Transpl Infect Dis.

[36] Sahin U, Toprak SK, Atilla PA (2016). An overview of infectious complications after allogeneic hematopoietic stem cell transplantation[J]. J Infect Chemother.

